# The Impact of Domestication on the Chicken Optical Apparatus

**DOI:** 10.1371/journal.pone.0065509

**Published:** 2013-06-12

**Authors:** Lina S.V. Roth, Olle Lind

**Affiliations:** 1 Department of Physics, Chemistry and Biology, Linköping University, Linköping, Sweden; 2 Department of Biology, Lund University, Lund, Sweden; Lund University, Sweden

## Abstract

Domestication processes tend to release animals from natural selection and favour traits desired by humans, such as food-production and co-operative behaviour. A side effect of such selective breeding is the alteration of unintended traits. In this paper, we investigate how active selection for egg production in chickens has affected the visual system, in particular the optical sensitivity that relates to the ability of chickens to see in dim light. We measured eye dimensions as well as the pupil diameter at different light intensities (the steady state pupil dynamics), in adult male and female White Leghorns and the closest relatives to their ancestor, the Red Junglefowls. With this information, we calculated the focal length and optical sensitivity (f-number) of the eyes. Males have larger eyes than females in both breeds and White Leghorn eyes are larger than those of Red Junglefowls in both sexes. The steady state pupil dynamics is less variable, however, the combination of pupil dynamics and eye size gives a higher optical sensitivity in Red Junglefowl eyes than in White Leghorns at light intensities below approximately 10 cd/m^2^. While eye size and focal length match the larger body size in White Leghorns compared to Red Junglefowls, the steady state pupil dynamics do not. The reason for this is likely to be that eye morphology and the neuro-muscular control of the pupil have been affected differently by the strong selection for egg production and the simultaneous release of the selection pressure for high performing vision. This study is the first description of how optical sensitivity has changed in a domesticated species and our results demonstrate important considerations regarding domestication processes and sensory ability.

## Introduction

Domestication changes the phenotype of animals according to active selection by humans [1], [2]. However, linkage between genes and pleiotropy can cause changes in traits other than those targeted for selection. The subject of this study, White Leghorn chickens, have been bred for maximum egg production and minimum food intake but in addition they have an adult bodyweight approximately double that of the wild ancestor, the Red Junglefowl [3].

Besides the active selection of desired traits, domestication processes alter the environmental conditions of animals by removing the threat of predation providing a rich access to food etc. The release of such constraints is likely to alter the selection pressure on sensory systems as well. As a consequence, the developmental, morphological and neurological features of the animal’s senses might change. An example of domestication effects has been reported by Dmitry K. Belyaevs who noticed that domesticated foxes responded to sound and opened their eyes earlier than wild-type foxes (reviewed in [2]).

Other indications of changes in sensory organs due to domestication come from investigations of the retinae in dogs and horses. McGreevy and colleagues [4] have reported differences in eye morphology between different dog breeds. Breeds with long noses have more pronounced horizontal visual streaks but less distinct area centralis than breeds with short noses. In horses, breeds with long noses have a higher density of ganglion cells in the horizontal visual streak than those with short noses while there is no correlation between skull morphology and cell density within the area centralis [5]. These retinal traits have not been actively selected for in domestication, but are better regarded as side effects from various breeding processes.

Just like horses and dogs, chickens are among the oldest domestic animals having been bred for egg laying or meat production for several thousands of years [6]. The effect of this long history of domestication on different traits in the chicken can be investigated by comparing traits of domestic animals with their progenitor, the Red Junglefowl (*Gallus gallus*) [6], [7]. There are indications of differences in temporal resolution in daylight between chicken breeds, ranging in CFFs (critical flicker fusion frequencies) from 71.5 Hz to 87 Hz [8]–[9] where the egg laying White Leghorn (*G. gallus domesticus*) is represented at the low end with 73.9 Hz (presented with numbers in [9] refering to original graph in [10]). Interestingly, the old game breed “Gammalsvensk dvärghöna”, which is similar to the wild-type ancestor the Red Junglefowl, has shown to have the highest CCFs of 87 Hz [9]. Lisney and colleagues [9] therefore suggest that temporal resolution in birds has been influenced by the artificial selection in breeding.

Is it possible that other visual functions, beside temporal resolution, have altered due to human selection as well? A major difference between the environmental constraints of Red Junglefowls and White Leghorns is the light conditions in which they search for food and interact with conspecifics and predators. While it is vital for Red Junglefowl chickens to maintain acute vision in the dim light of a thick understory, White Leghorns are less dependent on vision to express their behaviour in a predictable farm environment. At present, there are no reports of optical sensitivity in different chicken breeds and the effect from domestication on visual ability in dim light has been unknown.

The optical sensitivity of an eye (not considering photoreceptor sensitivity) is measured by the f-number, which is given by the pupil diameter divided by focal length. We might expect that eye size scales proportionally to the larger body size of White leghorns compared to Red Junglefowls, thus following the positive logarithmic relationship between body weight and eye axial length of birds in general [11]. As a consequence, focal length would be longer in White leghorn eyes than in Red Junglefowl. In contrast, it is more difficult to make predictions about differences in pupil size between the breeds since pupil dynamics depend on neuro-muscular control rather than anatomy, and it is not clear how this control might be affected by domestication.

In this study, we investigate eye morphology as well as pupil size at different light levels in White leghorns and Red Junglefowls and with this, we are able to calculate the optical sensitivity of the eyes as a function of light intensity. Our results provide valuable insights into how domestication processes and selective forces can affect visual ability.

## Methods

### Ethics Statement

All experiments in this paper were conducted in line with ethical approval from the regional ethical committee for animal experiments in Linköping, Sweden (Permit number: 122-10).

### Eye Anatomy and Focal Length

**Figure 1:**
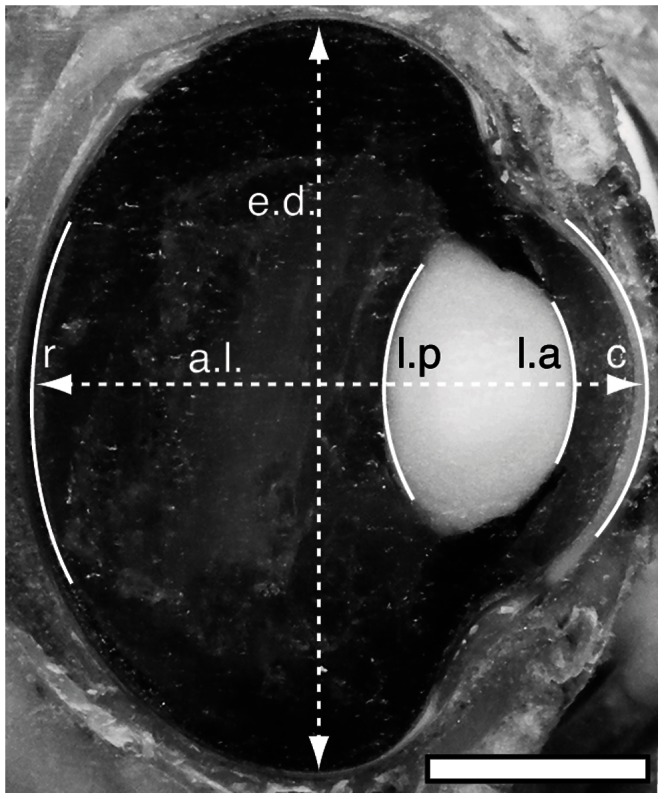
The right eye of a male Red Junglefowl. The eye is oriented so that the nasal region is upwards and the temporal region downwards in the image. The measured structures and distances are enhanced by white lines and indicated as; *a.l.* – axial length, *c* – cornea, *e.d.* – equatorial diameter, *l.a.* – lens anterior surface, *l.p*. – lens posterior surface, *r* – receptor layer. Anterior chamber depth is the distance between *c* and *l.a*., lens thickness is the distance between *l.a*. and *l.p*. and back vertex distance is the distance between *l.p*. and *r*. The scale bar is 5 mm.

White Leghorn chickens (originating from Scandinavian selection with a long history of selection for egg production, line SLU13; for details see [12]) and Red Junglefowl chickens (originated from a Swedish zoo population, for details see [13]) of both sexes were decapitated and heads were immediately frozen (−20°C). The eyes were embedded in a freezing medium (Tissue Tek OCT, Sakura Finetek Europe B.V., Alphen aan den Rijn, The Netherlands) and sagittally sectioned in a cryostat at a temperature of −10°C. Pictures were taken every 0.1 mm to obtain the largest eye dimensions (figure 1). At the largest eye dimension, we determined the positions and curvatures of the eye structures using ImageJ (version 1.43u; [14]). Distorted or damaged structures were not measured. Schematic eyes, following Land and Nilsson [15], were then constructed using the average eye parameters determined from the cryosectioning.

To estimate the refractive indices of the lenses for the schematic eyes we assumed emmetropic eyes with a refractive index of 1.337 for the aqueous and vitreous humour. From these assumptions, the bulk refractive index of the lens (assuming a homogenous equivalent to the gradient-index lens) was varied until the schematic eye agreed with the measured parameters (figure 1) in the real eyes [16]. The difference in axial lengths and other eye dimensions between sexes and breeds was tested with two-tailed two sample t-tests.

### Steady State Pupil Dynamics

The steady state pupil dynamics is a description of how the size of the pupil changes between light intensities for which the eye is sequentially allowed to fully adapt. We measured the pupil diameter under 7 different illuminations (0.00002, 0.016, 0.30, 1.7, 9.9, 26, and 177 cd/m^2^) in two males and two females of White Leghorn and Red Junglefowl. The different light levels were generated by indirect light (to create a homogenous light environment) from a combination of five halogen spots, two dimmable Dedolights (DLH4 universal 12/24 v tungsten lamp head with a spherics 2) and neutral density filters (LEE filters). Light intensity was measured in cd/m^2^ with a radiometer (ILT1700, International Light) as the reflection from a white standard at 45°. We did not make spectral measurements of the illumination. However, we used the same light source (Dedolights at their dimmest intensity), and thus the same illumination spectrum, for the light intensities between 0.00002 and 0.3 cd/m^2^. Similarly, the intensity change between 1.7 and 9.9 cd/m^2^ was acquired without spectral alteration using neutral density filters.

The birds were held by handler during the whole procedure to ensure that they were looking directly into the camera during the filming. The birds were allowed to adapt for 10 minutes at each light level before filming commenced. Three pictures of the pupils were captured at each light level (at approximately 40 s intervals) with an infrared sensitive video camera (Sony, HDR-CX11) and the largest diameter for each pupil picture was determined by using ImageJ [version 1.43u; 14]. All chickens used in this study were comfortable with being handled by humans and in the rare cases of observed symptoms of stress we allowed the birds to calm down before measurements were initiated.

### Optical Sensitivity

We calculated f-numbers as a function of light intensity by dividing the focal length in each sex and breed with the average pupil diameter throughout the intensity range. All calculations were performed using Matlab (version 7.12.0.635) and Microsoft Excel (version 14.2.4).

## Results

### Eye Anatomy and Focal Length

The axial lengths of male eyes were significantly longer than those of females (two sample t-test, p_White Leghorn_<0.02, p_Red Junglefowl_<0.02). For this reason, we compared the breeds for each sex separately in the analyses.

Axial length and equatorial diameter of the eyes in both male and female White Leghorns are larger than in Red Junglefowls (table 1) and White Leghorn males have larger anterior lens radii than Red Junglefowl males. In order to obtain the focal lengths in each sex and breed, we constructed schematic eyes using the measured parameters in table 1. The larger eyes in White Leghorns are matched by longer focal lengths in both sexes (table 1) and the ratio between focal length and axial length is similar in all groups (male White Leghorn, 0.65; male Red Junglefowl, 0.66; female White Leghorn, 0.62; female Red Junglefowl, 0.65).

**Table 1:** Eye Anatomy.

Male							
	White Leghorn			Red Junglefowl			
	average	SEM	*n*	average	SEM	*n*	twosample t-test
Corneal radius	5.31	0.1	5	5.06	0.12	7	n.s.
Anterior lens radius	5.59	0.24	5	4.33	0.19	6	**
Posterior lens radius	−4.65	0.42	4	−4.56	0.23	2	n.s
Lens thickness	4.21	0.05	4	4.05	0.16	3	n.s.
Anterior chamber depth	2.44	0.14	5	2.19	0.14	6	n.s.
Back vertex distance	8.36	0.24	4	7.75	0.05	5	n.s.
Axial length	14.95	0.12	5	13.78	0.06	5	***
Equatorial diameter	17.99	0.27	5	16.89	0.11	7	**
Focal length	9.73	–	–	9.12	–	–	–
Lens refractive index	1.470	–	–	1.462	–	–	–

Female							
	White Leghorn			Red Junglefowl			
	average	SEM	*n*	average	SEM	*n*	twosample t-test
Corneal radius	5.08	0.23	4	4.78	0.15	7	n.s.
Anterior lens radius	5.63	0.44	6	4.94	0.14	7	n.s.
Posterior lens radius	−4.59	0.49	3	−4.33	0.43	7	n.s.
Lens thickness	4.05	0.17	3	3.92	0.09	7	n.s.
Anterior chamber depth	2.08	0.22	4	1.88	0.05	7	n.s.
Back vertex distance	7.58	0.13	3	7.38	0.09	7	n.s.
Axial length	14.00	0.22	5	13.18	0.11	7	**
Equatorial diameter	17.34	0.22	6	15.70	0.11	7	***
Focal length	8.74	–	–	8.55	–	–	–
Lens refractive index	1.497	–	–	1.477	–	–	–

SEM stands for standard error of mean and *n* is the number of samples. Two sample t-tests (two-tail) were used to statistically compare the samples from each character and stars indicate the level of significance (**p<0.01, ***p<0.001). Focal length is given for the image side of the optical apparatus.

### Steady State Pupil Dynamics

**Figure 2:**
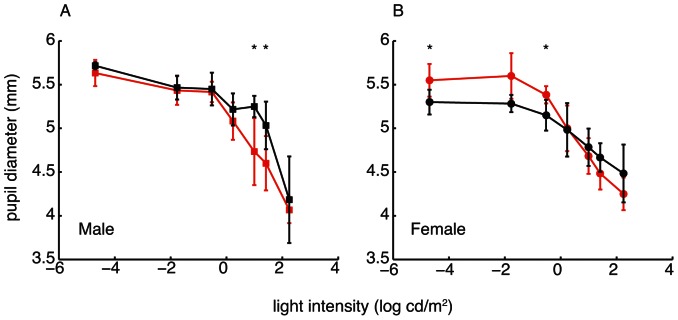
Steady state pupil dynamics of White Leghorn (black lines) and Red Junglefowl (red lines). Two males (A, squares) and two females (B, circles) were tested in each breed. Each point represents the average of six measurements in two individuals and error bars indicate the standard deviation. Stars indicate a significant difference between breeds (two sample t-test, p<0.05, two-tailed).

Pupil diameters of males and females of both White Leghorn and Red Junglefowl chickens were measured at different light intensities (figure 2). The steady state pupil dynamics of males are similar except at medium light intensities (9.9 cd/m^2^ and 25 cd/m^2^), where White Leghorns pupils are larger (figure 2). In females, the pupils are larger in Red Junglefowl at low light intensities (0.00002 cd/m^2^ and 0.3 cd/m^2^).

### Schematic Eye and Optical Sensitivity

**Figure 3:**
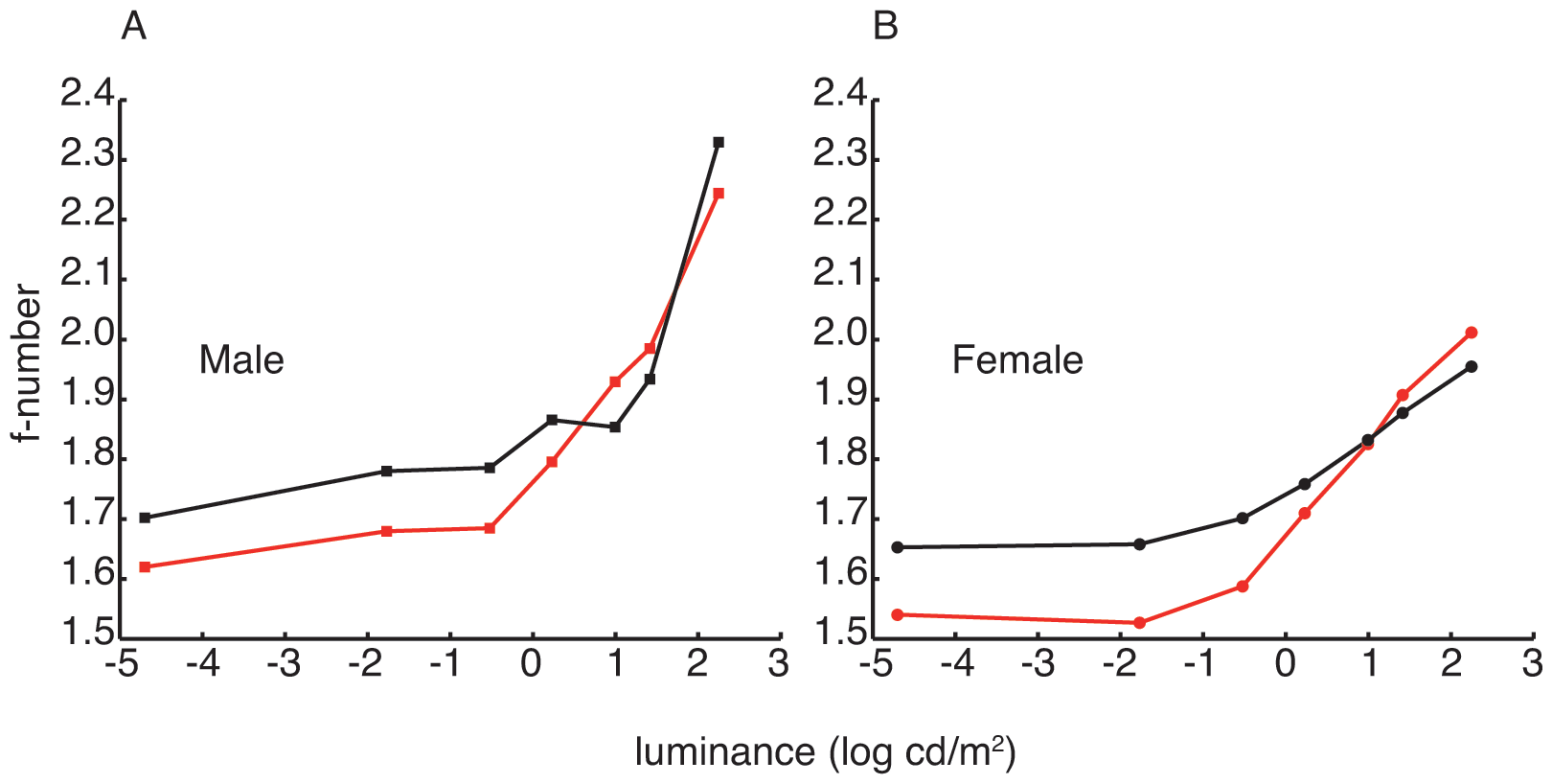
F-number as a function of light intensity in White Leghorn (black) and Red Junglefowl chickens (red). The f-number was calculated by dividing the focal length (table 1) with the pupil diameters at each intensity levels in males (A, squares) and females (B, circles).

The f-numbers decrease in dimmer light in all groups, though there is a larger change in males than in females (figure 3). There is little difference between White Leghorn and Red Junglefowl at higher light intensities while there is a distinct shift at lower light intensities below approximately 10 cd/m^2^ with markedly larger f-numbers in White Leghorns than in Red Junglefowls (figure 3). Hence, the eyes of White Leghorn chickens have a lower optical sensitivity than those of Red Junglefowls at low light intensities.

## Discussion

### Eye Size

This study is the first description of how optical sensitivity has changed in a domesticated animal. Our results show that the eyes in male chickens are larger than those in females and the eyes of both sexes are larger in White Leghorns than in Red Junglefowls (table 1). The ratios between focal length and axial length are, in all groups, within the range between 0.62 and 0.66 and there is only little variation in the refractive indices of the lenses that were adjusted for creating emmetropic schematic eyes (table 1). This strongly suggests that the optical structures in male and female chickens of different breeds generally increase with body size without substantial changes in proportionality.

### Steady State Pupil Dynamics and Optical Sensitivity

In contrast to eye size, we found only small differences in steady state pupil dynamics between breeds (figure 2). In dim light, the pupil diameters of males from the two breeds are similar, while Red Junglefowl females have larger pupils than female White Leghorns. As a result, both sexes of Red Junglefowl have more sensitive optics than White Leghorns at light intensities below approximately 10 cd/m^2^ (figure 3).

The optical sensitivity of the eye for a point light source is proportional to the squared pupil diameter while the sensitivity to an extended light source is proportional to the squared f-number [17]. At the dimmest light intensity (0.00002 cd/m^2^), male chickens from both breeds are optically equally sensitive to point light sources while female Red Junglefowls are about 8% more sensitive than female White Leghorns. For extended light sources at the same light intensity, the optics of Red Junglefowls of both sexes are between 11% and 15% more sensitive than White Leghorns.

In the light set up for the pupil measurements, the illumination spectrum might have changed slightly between some of the high light intensities (see methods). This is likely to be irrelevant since all birds experienced the same test conditions. Still, we cannot exclude the possibility that the spectral sensitivity of the pupil mechanism has been affected differently by domestication in the two breeds and that a small variability in the illumination spectrum of our experiments is of some importance for the results.

### Domestication Effects on the Chicken Visual System

Earlier studies have found a strong correlation between body size and egg production in White Leghorns [3]. Here we have found that White Leghorn chickens have larger eyes with less optical sensitivity than their progenitor, the Red Junglefowl. This difference is not due to unusually small eyes in Red Junglefowl, which have eye sizes comparable to other galliform birds living in similar enclosed habitats, such as the Ruffed grouse (*Bonasa umbellus,* eye axial length 13.73 mm) and the Spruce grouse (*Falcipennis* canadensis, eye axial length 13.25 mm; [18].

One explanation for the differences in eye size between White Leghorns and Red Junglefowl is that White Leghorns have increased in body size due to active selection for egg production and that eye size has followed this development. Interestingly, our results show that there is a difference in how eye morphology and neuro-muscular mechanisms are affected by breeding. Pupil size, which is given by a combination of muscular arrangement and neural control, is not larger in the larger eyes of White Leghorns compared to Red Junglefowls. As a consequence, White Leghorns have lost optical sensitivity as their eyes increased in size. Whether this is the result of physiological constraints or relaxed selection on visual capabilities remains to be tested.

In this study, we have investigated the optical sensitivity of the eyes. However, the visual sensitivity of behavioural responses depends on photoreceptor characteristics and post-receptor processes as well. Unfortunately, there are no comparative descriptions of these properties in White Leghorn and Red Junglefowl chickens. Likewise, it remains to be shown how the relatively large eyes and long focal lengths in White Leghorns (which potentially allow for a higher spatial resolution) relate to spatial vision. To answer these questions and further investigate the linkage between domestication and sensory ability, there is a need for morphological studies of retinal characteristics and behavioural tests of visual function.
